# The Role of Transient Receptor Potential Vanilloid 4 in Pulmonary Inflammatory Diseases

**DOI:** 10.3389/fimmu.2017.00503

**Published:** 2017-05-04

**Authors:** Rachel G. Scheraga, Brian D. Southern, Lisa M. Grove, Mitchell A. Olman

**Affiliations:** ^1^Cleveland Clinic, Department of Pathobiology, Lerner Research Institute, Cleveland, OH, USA

**Keywords:** transient receptor potential vanilloid 4, ion channels, asthma, pulmonary vascular disease, acute respiratory distress syndrome

## Abstract

Ion channels/pumps are essential regulators of organ homeostasis and disease. In the present review, we discuss the role of the mechanosensitive cation channel, transient receptor potential vanilloid 4 (TRPV4), in cytokine secretion and pulmonary inflammatory diseases such as asthma, cystic fibrosis (CF), and acute lung injury/acute respiratory distress syndrome (ARDS). TRPV4 has been shown to play a role in lung diseases associated with lung parenchymal stretch or stiffness. TRPV4 indirectly mediates hypotonicity-induced smooth muscle contraction and airway remodeling in asthma. Further, the literature suggests that in CF TRPV4 may improve ciliary beat frequency enhancing mucociliary clearance, while at the same time increasing pro-inflammatory cytokine secretion/lung tissue injury. Currently it is understood that the role of TRPV4 in immune cell function and associated lung tissue injury/ARDS may depend on the injury stimulus. Uncovering the downstream mechanisms of TRPV4 action in pulmonary inflammatory diseases is likely important to understanding disease pathogenesis and may lead to novel therapeutics.

## Introduction

Ion channels and pumps play multiple important roles in cell homeostasis (1). They function to allow passive, agonist-induced, or voltage-dependent flux of specific ions in and out of the cell (1, 2). Dysregulation of channel function and/or expression can lead to organ dysfunction and disease (1–3). Recent studies have shown that a transient receptor potential (TRP) channel family member, transient receptor potential vanilloid 4 (TRPV4), is implicated in inflammatory lung diseases such as asthma, cystic fibrosis (CF), acute lung injury/acute respiratory distress syndrome (ARDS), and pulmonary fibrosis (4–10). In fact, these studies show that TRPV4 can regulate inflammatory cytokines that play key roles in orchestrating lung tissue homeostasis and inflammatory lung disease (4, 7, 10–14). Dysregulation of cytokines leads to alterations in cell–cell interactions, lung tissue remodeling, and repair (15). Regulating cytokine secretion through the modulation of ion channels such as TRPV4 may mediate inflammatory lung diseases. Therefore, TRPV4 may be a potential target for lung disease pathogenesis (16). This review summarizes and integrates the data from our laboratory and others to further the understanding of the TRPV4–cytokine interaction in pulmonary inflammation.

## The TRPV4 Channel

Intracellular calcium is tightly regulated in a spatiotemporal manner through a system of ion channels and membrane pumps (17). One such channel is TRPV4, a transmembrane (TM) cation channel of the TRP superfamily (18). TRPV4 is an 871 amino acid protein that has 6 TM domains, an ion pore located between TM5 and 6, an NH_2_ terminal intracellular sequence with several ankyrin-type repeats, and a COOH-terminal intracellular tail (19, 20). Both the NH_2_ and COOH termini interact with signal kinases, other molecules [e.g., nitric oxide (NO)], and scaffolding proteins (21). The intracellular tails contain several activity-modifying phosphorylation sites. TRPV4 is sensitized and activated by both chemical [5,6-epoxyeicosatrienoic acid (EET) and 4 alpha-phorbol 12,13-didecanoate (4-αPDD)] and physical stimuli (temperature 27–35°C, membrane stretch, and hypotonicity) (22–25). TRPV4 is ubiquitously expressed in many cell types in the respiratory system. In the setting of pulmonary inflammation, TRPV4 has been found to be highly expressed and upregulated in airway smooth muscle, vascular endothelial cells, alveolar epithelial cells, and immune cells such as macrophages and neutrophils (12, 16, 21, 26–28). TRPV4 has been implicated in the pathogenesis of asthma, CF, and sterile and infection-associated ARDS (4–10, 29).

## The Role of TRPV4 in Inflammatory Lung Diseases

### Asthma

Asthma is a chronic lung disease characterized by airway inflammation and remodeling, excess bronchial secretions, and smooth muscle hypertrophy and contraction leading to airway narrowing (bronchoconstriction). Recent work shows that TRPV4 mediates airway wall thickness, goblet cell recruitment, collagen expression, fibrotic airway remodeling, and increased expression of transforming growth factor-β (TGF-β) in a house dust mite (*Dermatophagoides farinae*) mouse model of asthma (30). The authors also show that TRPV4 mediates TGF-β-dependent myofibroblast differentiation *in vitro* through the ras homolog gene family member A (RhoA), p38, and PI3Kα (30). *In vitro* exposure of airway smooth muscle or tracheal rings to hypotonic solutions causes smooth muscle cell contraction, and some asthmatic patients are hypersensitive to this stimulus. To that end, it has been found that small nucleotide polymorphisms in the G allele in the coding region and 3′ flanking region of the TRPV4 gene, as first identified in COPD, are associated with a greater reduction in pulmonary function after hypotonic saline administration (8, 31). Interestingly, the calcium and contractile response of smooth muscle cells to hypotonic saline involves interactions between the cysteinyl leukotriene pathway and TRPV4 (12, 32). These findings suggest that downregulation of TRPV4 may be a therapeutic target in some etiologies and genetic variants of asthma. Of note, different TRPV4 activation stimuli beyond hypotonicity utilize different pathways for TRPV4 activation. For example, hypotonicity induces TRPV4 activation through phospholipase A2 (PLA2)/P450 epoxygenase-dependent generation of EETs, while heat and 4αPDD are PLA2/P450-independent (25). Further study of the mode of TRPV4 activation in individual diseases would support disease-specific, pathway-targeted therapy. While asthma is an inflammatory disease, there is no current evidence linking Th2-type cytokines and TRPV4 in the pathogenesis of asthma. Hence, this is an avenue for future studies.

### Cystic Fibrosis

Cystic fibrosis is characterized by a mutation in the cystic fibrosis transmembrane conductance regulator (CFTR), a membrane-based chloride channel, which initially causes dehydration of the airway surface liquid thereby increasing susceptibility to bacterial and fungal infections (e.g., *Pseudomonas, Staphylococcus, Burkholderia*, atypical mycobacterium) (33). TRPV4 interacts with CFTR on several levels. TRPV4-dependent calcium influx in response to hypotonicity is reduced in human CF epithelial cells (34). Furthermore, other hypotonicity-induced TRPV4 chemical activators (5,6-, 8,9-, 11,12-, and 14,15-EET) and their metabolites (5,6 DHET) have been measured in the sputum of CF patients (10). Although the current consensus suggests that dehydration of airway mucous is the predominant cause of impaired mucociliary clearance in CF, recent considerations have been put forth to increase ciliary function or ciliary beat frequency (CBF) as a means to improve mucociliary clearance (35, 36). Concordantly, TRPV4-deleted tracheal epithelial cells have decreased CBF in response to ATP, 4αPDD, and temperature, whereas CBF in response to hyperviscosity was similar in wild-type (WT) and TRPV4 deleted cells. These data suggest that TRPV4 agonism might increase CBF; however, the effects on CF prognosis remain to be determined (37).

The pathogenesis of CF is also characterized by cytokine-mediated airway inflammation. Recently, both cytokines/chemokines and lipid mediators secreted from epithelial cells have been identified as key components in the inflammatory process. In this regard, TRPV4 activation induces epithelial cell secretion of pro-inflammatory cytokines/chemokines and active lipid mediators (e.g., IL-8, cytosolic PLA2, prostaglandin E2, NF-κB, AA, etc.) in response to lipopolysaccharide (LPS) (10). Secretion of IL-8/KC, in both bronchial epithelial cells and in intact mice lungs in response to TPRV4 activation, was increased upon inhibition of CFTR (10). These data demonstrate that TRPV4 has pleotropic effects on CF pathogenesis. Further study of the individual molecular pathways downstream of TRPV4 in CF may identify selectivity in the TRPV4 responses that can then be marshaled for therapeutic intent.

## Acute Lung Injury/ARDS

Acute respiratory distress syndrome is a syndrome characterized by patchy lung inflammation along with cytokine release leading to alveolar space edema, exudate, and collapse. The pathogenesis of ARDS is complex; it is characterized by endothelial and alveolar epithelial injury followed by recruitment and accumulation of inflammatory cells in the injured alveolus (38). ARDS is a consequence of non-infectious (trauma, hemorrhage, lung ventilator stretch) or infectious (sepsis, pneumonia) causes (39). As the biological processes that underlie the lung injury and their molecular drivers are not fully understood, medical therapy directed at the lung inflammatory response has yet to successfully modify the course of ARDS. Experimental animal and patient studies demonstrate the lung injury and resolution phases of ARDS are mediated through a complex orchestration of cytokines/chemokines (e.g., IL-1β, TNFα, IL-8, IL-6, and IL-10) (40–44). Studies show that both sterile (e.g., ventilator-induced stretch) and infectious [e.g., intra-tracheal (IT) LPS] triggers of ARDS result in stiffening (reduced compliance) of the lung tissue (45, 46).

The role of TRPV4 in ARDS is context/etiology-dependent. It has been shown that TRPV4 mediates the lung injury response to a sterile stimulus *in vivo* [i.e., hydrochloric acid (HCl)], as assessed by inflammatory cell influx, lung vascular permeability (wet/dry ratio, Evans blue dye extravasation, and total protein), lung histopathology and physiology, and pro-inflammatory cytokine levels (IL-1β, VEGF, KC, G-CSF, MCP-1, RANTES, MIP-2, and IL-6) (7, 14). Protection from the acute lung injury response to IT HCl was noted in mice that lack TRPV4 (TRPV4 KO), or in mice that were treated with three different small molecule inhibitors of TRPV4 (7, 14). Importantly, two of these inhibitors (GSK2220691 and GSK2337429A) show efficacy when administered 30 min after IT HCl (7). Thus, these inhibitors show promise as a novel and exciting therapeutic/preventative approach for acute lung injury (7). *In vitro* stimulation of human and murine neutrophils (with platelet-activating factor or LPS) induced TRPV4-dependent calcium influx, reactive oxygen species (ROS) production, adhesion chemotaxis, and Rac activation (14). Taken together, these data suggest that neutrophils possess the capacity to mediate acute lung injury in a TRPV4-dependent manner. Whether the *in vivo* lung injury response to HCl is solely dependent on neutrophil TRPV4, as opposed to TRPV4 in other cell types, remains to be determined. In addition to TRPV4’s effect on the cytokine/inflammatory changes in ARDS, TRPV4 actions can induce lung endothelial barrier dysfunction *in vitro* and *in vivo*, as well as cause disruption of alveolar type I epithelial cells leading to lung vascular leak and alveolar edema (9, 29). These findings are the rationale for a clinical trial of TRPV4 antagonists in high venous pressure-induced pulmonary edema (https://clinicaltrials.gov).

## TRPV4 and Macrophage Function in Lung Injury

A similar TRPV4-dependent lung injury response has been demonstrated in macrophages in high volume ventilator-induced lung injury (6, 47). Mice lacking TRPV4 (TRPV4 KO) had less vascular leak, pulmonary edema (wet/dry ratio, filtration coefficient), and NO production in response to high volumes (peak inflation pressure 35 cm H_2_O) when compared to WT controls. TRPV4 also seemed to partially mediate the increase in injury due to the combined effects of high volume ventilation and induced hyperthermia (40°C). Analysis of alveolar macrophages after high volume ventilation revealed that TRPV4 KO macrophages had less production of NO and ROS than those from WT mice. As in the HCl model, pretreatment with a non-selective TRP inhibitor (ruthenium red) prevented the increase in vascular permeability from combined high volume ventilation/hyperthermia in WT mice (48). Adoptive transfer of WT macrophages to TRPV4 KO mice reestablished the lung injury seen in WT mice. These data suggest that macrophage-specific TRPV4 acts as a mechanical and temperature sensor to initiate/mediate the acute lung injury induced by high volume ventilation (47).

Our laboratory is studying the role of TRPV4 in macrophage function during infection-associated lung injury. Alveolar macrophages are known to be effector cells in bacterial and particle clearance, and in the injury/repair process (49). We chose to explore the role of the calcium ion channel, TRPV4, in macrophage phagocytosis, as intracellular calcium is known to be required for the phagocytic process, and because TRPV4 plays a role in force-dependent cytoskeletal changes in other systems/cell types (7–9, 29, 47, 50, 51). Studies show that the process of phagocytosis in macrophages requires integration of the signals from macrophage surface receptors, pathogens, and the extracellular matrix (52–54). However, the effects of matrix stiffness on the macrophage phenotypic response or its signal transduction pathways have yet to be fully elucidated.

We recently published the novel observation that TRPV4 integrates the LPS and matrix stiffness signals to control macrophage function, which promotes host defense and resolution from lung injury (4). After demonstrating that TRPV4 is expressed and functionally active in murine bone marrow-derived macrophages, we studied the macrophage response to LPS on matrices of varying physiological-range stiffnesses. We demonstrated that TRPV4 mediates LPS-stimulated macrophage phagocytosis of both opsonized particles (IgG-coated latex beads) and non-opsonized particles (*Escherichia coli*) *in vitro*. Matrix stiffness in the range seen in inflamed or fibrotic lung (>25 kPa) augmented the LPS phagocytic response by 151 ± 3% (4). Inhibition of TRPV4 by siRNA or pharmacologic inhibitors completely abrogated both the LPS effect, as well as the matrix stiffness effect, on phagocytosis. These data indicate that both the LPS and stiffness effect on macrophage phagocytosis are TRPV4 dependent (4).

**Figure 1:**
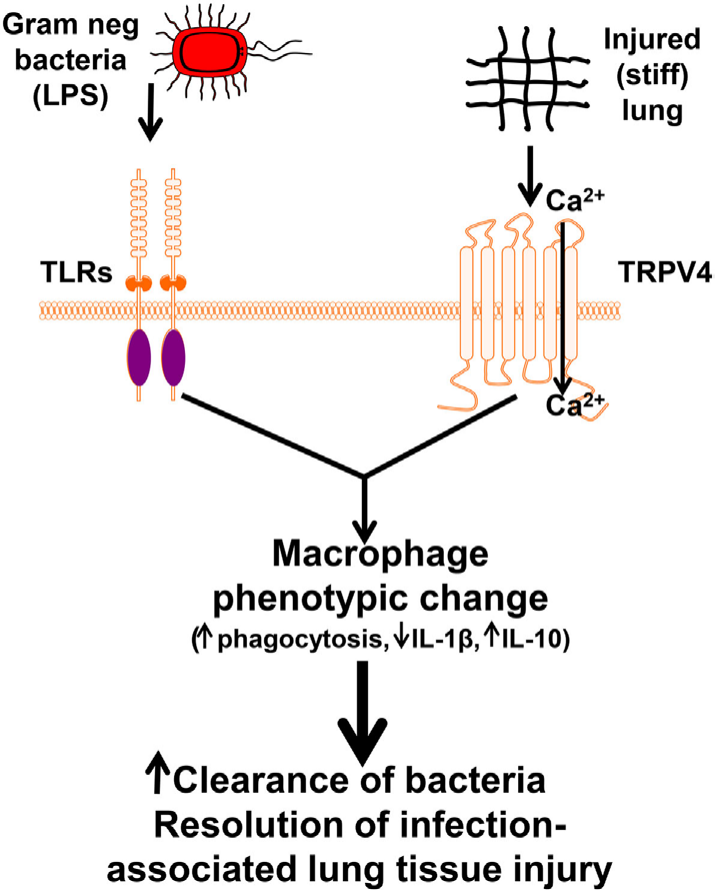
**Working model illustrating that lipopolysaccharide (LPS) and transient receptor potential vanilloid 4 (TRPV4) signal cooperate to alter macrophage phenotypic change leading to enhanced clearance of bacteria and resolution of lung injury**. Our data suggest that TRPV4 is sensitized by extracellular matrix stiffness in the range of inflamed/fibrotic lung. Interaction between the LPS signal and the matrix stiffness signal through TRPV4 promote increased TRPV4 channel activity and macrophage phenotypic change leading to increased clearance of bacteria and resolution of infection-associated lung injury (4). Copyright 2016, The American Association of Immunologists, Inc.

As TRPV4 is required for macrophage phagocytosis *in vitro* in a stiffness-dependent manner, we next sought to examine the role of TRPV4 on macrophage phagocytosis after intratracheally (IT) administered LPS *in vivo*. Despite the influx of neutrophils, alveolar macrophages were the predominant cell type that phagocytosed IT administered IgG-coated beads following IT LPS (24 h) in WT mice (4). As seen *in vitro*, the *in vivo* enhancement effect of IT LPS on alveolar macrophage phagocytosis was lost upon deletion of TRPV4 (TRPV4 KO mice) (Figure 1) (4). This effect is not explained by a difference in macrophage recruitment. Concordant with the *in vitro* data, our *in vivo* data demonstrate that LPS-induced alveolar macrophage phagocytosis is TRPV4 dependent.

Studies suggest that macrophage-released cytokines modulate bacterial clearance and the lung injury/repair process, in the context of injury-related stiffened matrix (52–55). Recognizing the complexity of tissue responses to individual cytokines/chemokines, we chose to focus initially on IL-1β and IL-10, as they are well-known key mediators of lung injury/resolution (56–58). TRPV4 also modulates the LPS signal for cytokine production. Specifically, IL-1β secretion was decreased by half, and IL-10 secretion increased approximately twofold in WT alveolar macrophages compared with TRPV4 KO macrophages in response to LPS. Such a profile would predict that TRPV4 mediates a net inflammation-suppressive response to LPS. Interestingly, this TRPV4 modulation of the LPS signal required a matrix stiffness in the range of injured or fibrotic lung (≥25 kPa). As illustrated in the schematic model, macrophage TRPV4 is sensitized by a stiff matrix (as seen in ARDS) to modulate the infectious (LPS—experimental surrogate for Gram-negative bacterial lung infection) signal toward an anti-inflammatory macrophage phenotype (Figure 1).

Collectively, our data demonstrate that TRPV4 responds to extracellular matrix stiffness, thereby altering the LPS signal to mediate macrophage phagocytosis and cytokine production (4). Despite the limitations in extrapolating our simplified experimental system to *in vivo* lung injury, the data point to TRPV4 as an important mechanosensor that mediates macrophage function differently in lung homeostasis, and in the context of pulmonary infection-induced inflammation. We speculate that under basal conditions, the resident lung macrophage response to LPS is modified (less phagocytic, more IL-1β) as a consequence of low lung tissue stiffness (i.e., 1–3 kPa) thereby enhancing recruitment of professional bactericidal cells (neutrophils) (55). After an acute inflammatory or infectious insult, a separate population of monocytes is recruited from the bone marrow to populate both interstitial and injured alveolar compartment in the context of denuded, exposed interstitial matrix (40). There are two overlapping phases of ARDS. During the initial injury phase (days 1–10), lung tissue is predominantly edematous and exudative, while during the fibroproliferative phase (days 7–28), there is increased deposition of interstitial and alveolar type I and III collagen (40). Both phases of ARDS (fibroproliferative > acute) exhibit clear evidence of increased stiffness at the whole organ level (40, 46, 59), but, limited mechanical data are available at the cellular level of resolution. A recent study shows that lung alveolar vessel wall stiffness is increased >10-fold (3 versus 43 kPa) after IT LPS (48 h) in mice compared to controls, as measured by atomic force microscopy, well within the range examined in our study (>8–25 kPa) (45, 46). We further speculate that, after injury, the macrophage phagocytic response to LPS is upregulated along with a cytokine profile that promotes resolution in a TRPV4-dependent manner as a consequence of tissue stiffening. Such a scenario would support tissue stiffness, TRPV4-dependent shift in the macrophage phenotype that is commensurate with the appropriate phase of the injury/repair process.

Thus, our findings suggest that TRPV4 regulates a feed-forward mechanism of phagocytosis in activated lung tissue macrophages when they interact with stiffened infection/injury-associated lung matrix. This concept is further supported by the observation that surfactant protein B-deficient mice have altered alveolar macrophage shape and function in association with increased alveolar surface tension (60). The macrophage activation phenotypes (M1/M2 classification) are well established *in vitro*. The classically activated M1 macrophage phenotype, induced by INFγ, TNFα, and LPS, exhibits inflammatory/bactericidal properties. In contrast, the alternatively activated M2 macrophage phenotype, induced by IL-4 and IL-13, exhibits tissue repair/fibrotic properties (49, 55). Data are emerging that the *in vivo* macrophage phenotypes are more heterogeneous and plastic than the *in vitro* derived M1/M2 classification. Our published cytokine data (↑IL-1β, ↓IL-10) with inhibition of TRPV4 indeed suggests that TRPV4 mediates polarization toward M1-like phenotype (4, 61, 62). However, a complex array of cytokines contributes to the pathogenesis of ARDS, and targeting individual cytokines has not been shown to alter the disease process, indicating the net inflammatory balance is important (41–44, 63).

Our findings regarding the role of TRPV4 in downregulating the pro-inflammatory, bacterial clearance-inducing LPS signal are opposite to those in neutrophils in response to sterile inflammation, or in macrophages upon stretch-induced tissue injury. Lung injury is dependent on cytokine production and inflammatory cell influx in response to activation of pattern recognition receptors by damage-associated molecular patterns (DAMPs) and pathogen-associated molecular patterns (PAMPs). There are multiple known ligand–receptor interactions and intracellular signaling pathways that are both DAMP/PAMP-receptor specific and overlapping. We speculate that differences in the interaction of TRPV4 signals with infectious PAMP signals versus sterile tissue injury DAMP signals might explain the differences between our infectious model and the sterile lung injury model. Defining the specific molecular pathways and interactions in individual injury models is a fruitful avenue of research that may lead to novel therapeutic targets.

## Summary

In summary, ion channels are important in the pathogenesis of inflammatory lung diseases, and the ion channel TRPV4 plays a specific role in mediating lung diseases associated with parenchymal stretch and inflammation or infection. The data reviewed in this work on the role of TRPV4 in pulmonary inflammatory diseases are summarized in Table 1. TRPV4 activation and its downstream signaling pathways differ in response to varying stimuli, cell types, and contexts. In asthma, TRPV4 mediates hypotonicity-induced airway hyperresponsiveness, but not release of Th2 cytokines (12, 32). In CF, TRPV4 appears to play important, yet paradoxical, roles in CBF/mucociliary clearance and epithelial cell pro-inflammatory cytokine (IL-8/KC) secretion (35, 36). TRPV4 may also play different roles in ARDS depending on the underlying etiology (4, 7, 14, 48). We, and others, have shown that macrophage and neutrophil TRPV4 regulate pro-inflammatory cytokine secretion. Lastly, in pulmonary fibrosis, TRPV4 has been shown to mediate the mechanosensing that drives myofibroblast differentiation and experimental lung fibrosis in mice (5). Collectively, TRPV4 is shown to play a novel role in modulating cytokine secretion and pulmonary inflammation and therefore may be involved in the pathogenesis of many respiratory diseases.

**Table 1:** ***In vitro* and *in vivo* studies of the role of transient receptor potential vanilloid 4 (TRPV4) in inflammatory pulmonary diseases**.

Disease	Cell type	Key findings	Reference
Asthma	Fibroblasts	Transforming growth factor-β-dependent airway remodeling	(30)

	Smooth muscle cells	Hypotonicity-induced calcium and contractile response	(12, 32)

Cystic fibrosis (CF)	Epithelial cells (tracheal and airway)	Regulates ciliary beat frequency	(10, 33–37)
		Decreased ATP-induced calcium influx	
		Pro-inflammatory cytokine production (e.g., IL-8, cytosolic PLA2, prostaglandin E2, NF-κB, arachidonic acid, etc.)	

Acute lung injury/acute respiratory distress syndrome (ARDS)	Epithelial cells	Maintains epithelial barrier function	(9, 29)

	Endothelial cells	Maintains endothelial septal barrier	(11)

	Neutrophils	Calcium influx	(7, 14)
		Reactive oxygen species production	
		Adhesion chemotaxis	
		Rac activation	

	Macrophages	Lipopolysaccharide-induced macrophage phagocytosis *in vitro* and *in vivo*	(4, 6, 47, 48)
		Anti-inflammatory cytokine production (IL-1β, IL-10)	

Pulmonary fibrosis	Fibroblasts	Myofibroblast differentiation	(5)
		Experimental pulmonary fibrosis in mice	

*This table is only a partial representation of the literature, given the focused nature of the mini review. We apologize for any work omitted from this review. We summarize the cited literature on the role of TRPV4 in asthma, CF, acute lung injury/ARDS, and pulmonary fibrosis*.

## Author Contributions

RS, BS, LG, and MO reviewed the literature and wrote the paper.

## Conflict of Interest Statement

The authors declare that the research was conducted in the absence of any commercial or financial relationships that could be construed as a potential conflict of interest.
